# Exploring the potential of ChatGPT as a supplementary tool for providing orthopaedic information

**DOI:** 10.1007/s00167-023-07529-2

**Published:** 2023-08-08

**Authors:** Janina Kaarre, Robert Feldt, Laura E. Keeling, Sahil Dadoo, Bálint Zsidai, Jonathan D. Hughes, Kristian Samuelsson, Volker Musahl

**Affiliations:** 1 Department of Orthopaedic Surgery, UPMC Freddie Fu Sports Medicine Center, University of Pittsburgh, Pittsburgh, USA; 2 Department of Orthopaedics, Institute of Clinical Sciences, Sahlgrenska Academy, University of Gothenburg, Göteborgsvägen 31, 431 80 Mölndal, Sweden; 3 Department of Computer Science and Engineering, Chalmers University of Technology, Gothenburg, Sweden; 4 Department of Orthopaedics, Sahlgrenska University Hospital, Mölndal, Sweden

**Keywords:** Large language models, ChatGPT, Anterior cruciate ligament, ACL, Artificial intelligence, Correctness

## Abstract

**Purpose:**

To investigate the potential use of large language models (LLMs) in orthopaedics by presenting queries pertinent to anterior cruciate ligament (ACL) surgery to generative pre-trained transformer (ChatGPT, specifically using its GPT-4 model of March 14th 2023). Additionally, this study aimed to evaluate the depth of the LLM’s knowledge and investigate its adaptability to different user groups. It was hypothesized that the ChatGPT would be able to adapt to different target groups due to its strong language understanding and processing capabilities.

**Methods:**

ChatGPT was presented with 20 questions and response was requested for two distinct target audiences: patients and non-orthopaedic medical doctors. Two board-certified orthopaedic sports medicine surgeons and two expert orthopaedic sports medicine surgeons independently evaluated the responses generated by ChatGPT. Mean correctness, completeness, and adaptability to the target audiences (patients and non-orthopaedic medical doctors) were determined. A three-point response scale facilitated nuanced assessment.

**Results:**

ChatGPT exhibited fair accuracy, with average correctness scores of 1.69 and 1.66 (on a scale from 0, incorrect, 1, partially correct, to 2, correct) for patients and medical doctors, respectively. Three of the 20 questions (15.0%) were deemed incorrect by any of the four orthopaedic sports medicine surgeon assessors. Moreover, overall completeness was calculated to be 1.51 and 1.64 for patients and medical doctors, respectively, while overall adaptiveness was determined to be 1.75 and 1.73 for patients and doctors, respectively.

**Conclusion:**

Overall, ChatGPT was successful in generating correct responses in approximately 65% of the cases related to ACL surgery. The findings of this study imply that LLMs offer potential as a supplementary tool for acquiring orthopaedic knowledge. However, although ChatGPT can provide guidance and effectively adapt to diverse target audiences, it cannot supplant the expertise of orthopaedic sports medicine surgeons in diagnostic and treatment planning endeavours due to its limited understanding of orthopaedic domains and its potential for erroneous responses.

**Level of evidence:**

V.

**Supplementary Information:**

The online version contains supplementary material available at 10.1007/s00167-023-07529-2.

## Introduction

During the past few months, large language models (LLMs), such as generative pre-trained transformer (ChatGPT), have garnered significant attention, making them one of the most highly discussed topics worldwide. Furthermore, ChatGPT has recently demonstrated remarkable abilities in achieving excellent performance in the United States Medical Licensing Examinations (USMLE) as well as American Board of Neurological Surgery (ABNS) rxaminations, which assess comprehensive and detailed medical knowledge [4, 15]. Despite their potential, LLMs have also generated controversy [20, 21], as scientists have expressed concerns about potential threats to scientific transparency as well as misinformation leading to ethical concerns, such as posing risks to health and equity [3, 22]. Nevertheless, as the potential applications of ChatGPT are considerable, it has become one of the most popular artificial intelligence (AI) tools available.

Despite the growing interest in implementing LLMs in medical research [10, 11, 16, 24], there is a lack of discussion on the correctness, completeness, and adaptability (to different target groups) of the responses provided by these models, in particular within sports medicine and orthopaedics. Thus, while LLMs, such as ChatGPT, offer significant potential for delivering concise medical information, there also exists the possibility of providing patients with inaccurate information. [5–7, 14, 22, 24]. Therefore, the aim of this study was to investigate the feasibility of utilizing LLMs in orthopaedics by posing to ChatGPT questions relevant to anterior cruciate ligament (ACL) surgery and evaluating its responses by orthopaedic sports medicine surgeons in the field. Additionally, this study aimed to evaluate the depth of the LLM’s knowledge (correctness and completeness) and investigate its adaptability to different user groups (patient and non-orthopaedic medical doctor). It was hypothesized that the ChatGPT would be able to adapt to different target groups and provide generally good responses due to its strong language understanding and processing capabilities.

## Material and methods

### Data source

To identify high-yield questions relevant to ACL surgery, a thorough literature search was conducted and consensus statements in the field were reviewed [9, 18]. To generate inclusiveness, questions that are frequently asked by patients in clinical settings were also used. These questions were subsequently modified to feature simple syntax and grammar. The questions were additionally modified to be short enough to allow for succinct responses. A total of 20 questions were selected and included in the current study (Supplemental material).

### ChatGPT

ChatGPT is a type of LLM based on a transformer-style neural network architecture that is pre-trained on a large corpus of text to predict the next token in a document [17]. It was first introduced as a research variant in November 2022 [2]. However, a new version of ChatGPT, using GPT-4 as the underlying model, has been launched already in March 2023 [1] and exhibits the ability to provide responses that are human-like as well as demonstrates early signs of general intelligence [8]. Thus, this model (GPT-4 of March 14th 2023) was used in this study.

### Prompting and response collection

It is known that the method of prompting LLMs like ChatGPT can significantly impact on the quality of their responses; thus, a sub-field of study called ‘Prompt Engineering’ has been developed to provide advice on this craft [13, 23]. Therefore a prompt in line with these guidelines was created, to provide a proper setting for the model to answer the questions to the best of its abilities. Specifically, the model was asked to be an expert orthopaedic surgeon and to answer based on the latest research and best practices. Detailed instructions about the target group and what the model could expect them to know were included, as well as detailed guidelines on the expected form of response (Table 1). The length of responses was limited to avoid risks during assessments, e.g. that our assessors would not be able to locate the core answer in a long response. A shorter response would also induce the model to include more relevant information. However, for the target group of medical doctors, we allowed for a longer response (maximum 7 instead of 5 sentences), since it was anticipated that the use of more precise terms and concepts would lengthen responses. The two prompts used can be seen in Table 1; they share the same prefix and suffix but otherwise differ. As can be seen, the model in zero-shot mode was used, i.e. without providing examples of the type of questions we would pose and the answers we expected. This is a more challenging, but, arguably, also more realistic usage mode than the multiple-choice or few-shot setting of several other benchmarks [15].

**Table 1:** Table illustrating the two prompts used in this study

I want you to act as an English-speaking orthopaedic surgeon specializing in sports medicine and knee ligament surgery. Not only are you a practising surgeon, you are also up to date with the latest research in the field; base your work on it, and run research studies yourself to further advance the field. Your task is to answer questions about knee injuries and the treatment options. I will write the questions to you and you will answer based on the latest, state-of-the-art orthopaedic knowledge and on current established standards for treatment. Your answer must be adapted to the target group	
Target group “patient, young athlete”: The target group is a patient that is an adult, a young athlete, that has a high school degree but no specific medical training or experience. Your answers need to be understandable and rather brief, preferably 2–3 sentences and not longer than 5 sentences. Don't use overly complex language or wording: the goal is to be clear, direct, and understandable You cannot assume the patient has deep knowledge of anatomy or physiology, nor about the jargon or specific terms of the field, but you can assume that the patient has a basic understanding of the human body and its functions	Target group “medical doctor”: The target group is a medical doctor that has knowledge of anatomy and physiology and a basic understanding of surgical procedures but has no deeper knowledge about surgery or about the specific treatment options and their relative merits. Your answers need to be precise but rather brief, preferably 2–3 sentences and not longer than 7 sentences You can use complex language and wording: the goal is to be precise, give expert advice, and provide a broad sense of multiple treatment options. Your answers should be as complete as possible and not leave out any of the important factors. You can assume that the medical doctor has knowledge of anatomy and physiology and a basic understanding of surgical procedures but has no deeper knowledge about (knee) surgery nor about the specific treatment options and their relative merits.
I want you to only reply with your answer, nothing else My first question is “[QUESTION]”	

The order of the questions was randomized to negate any potential systemic effects of context and order on the answers given. The same random order was used for the two target groups. After the initial prompt and the response, the response was copied and then prompted again in the format ‘My next question is “[QUESTION]”’ until all questions of the sequence had been responded to.

After collecting all responses, an online questionnaire per target group was created, which listed the questions and responses to enable assessors to rate the correctness, completeness, and adaptiveness to the target group. Assessors were also permitted to add comments to explain their choices. Detailed instructions were provided that included examples of how to judge the different criteria. Each assessor was then provided with the instructions and links to their two questionnaires. The assessments of all four assessors were extracted and summarized.

### Assessment

Review and assessment of the responses provided by ChatGPT were performed independently by two board-certified orthopaedic sports medicine surgeons and two expert orthopaedic sports medicine surgeons in the field. The correctness was graded as 0 = incorrect; 1 = partially correct and 2 = correct, while completeness was graded as 0 = incomplete; 1 = partially complete and 2 = complete. Finally, adaptiveness (to the target group) was graded as 0 = not adapted; 1 = somewhat adapted and 2 = well adapted. Any discrepancies in assessment made by the four orthopaedic sports medicine surgeons/professors were subjected to discussion and commentary by the two expert professors within the field. The goal was not to decide on a final, overall judgement per response, but rather to better understand the reasons for different judgements; this could better reflect the nuance that may be involved in answering state-of-the-art questions in any scientific field. Tables 2 and 3 thus report the initial grading of each assessor, sorted from higher values to lower.

**Table 2:** Overall summary for all the responses

Target	Mean correctness	Mean completeness	Mean adaptiveness	Mean completeness*
Patient	1.69	1.51	1.75	1.53
Medical Doctor	1.66	1.64	1.73	1.65

^*^Mean completeness of responses with a mean correctness score ≥ 1.5 and without receiving any score of “0” (incorrect) was also calculated

**Table 3:** Detailed information on responses to questions for target group “patient, young athlete”

Label	Num questions	% questions	Mean correctness	Correctness grading	Example question	ChatGPT response
Fully correct	4	20.0	2.0	2,2,2,2	What strategies should be used to counteract kinesophobia?	To counteract kinesophobia (fear of movement or re-injury), patients should gradually expose themselves to activities under professional guidance, such as physical therapy. Setting realistic goals, receiving education about the injury and recovery process, and using psychological techniques like relaxation or visualization can also help manage fear and build confidence.
Majority correct	9	45.0	1.75	2,2,2,1	What are the most important risk factors for postoperative knee stiffness following anterior cruciate ligament reconstruction?	Key risk factors for postoperative knee stiffness after ACL reconstruction include preoperative knee stiffness, prolonged immobilization, incorrect graft placement, and inadequate rehabilitation Additionally, factors like infection, bleeding, or an individual's natural healing response can contribute to stiffness. Proper surgical technique and a well-designed rehabilitation plan can help minimize these risks.
Correct/partial	4	20.0	1.5	2,2,1,1	What are the risks and benefits of stump preservation in anterior cruciate ligament reconstruction?	Stump preservation in ACL reconstruction aims to maintain the remaining ACL tissue to potentially improve stability and healing. Benefits include better knee proprioception and faster recovery. However, risks involve possible graft impingement, inadequate tunnel placement, or complications in revision surgeries. A surgeon will weigh these factors when deciding on the best approach.
Correct/diverging	1	5.0	1.5	2,2,2,0	When should a lateral augmentation procedure be added to an anterior cruciate ligament reconstruction?	A lateral augmentation procedure, like a lateral extra-articular tenodesis, should be considered in ACL reconstruction when there is significant rotational knee instability, a high risk of graft failure (e.g., young athletes in pivoting sports), or a history of failed primary ACL reconstruction. It can help improve overall knee stability and reduce the risk of re-injury
Partially correct/diverging	2	10.0	1.25	2,2,1,0	What are the indications for anterior cruciate ligament injuries in children and when should they be treated?	Indications for treating ACL injuries in children include significant knee instability, a desire to return to high-level sports, or associated injuries like meniscus or other ligament tears. Treatment options range from conservative management with bracing and physical therapy to surgical intervention, such as ACL reconstruction. The timing of treatment depends on the severity of the injury, the child's skeletal maturity, and individual factors discussed with the surgeon.

### Equity, diversity, and inclusion

This study included orthopaedic sports medicine conditions that are relevant to patients of both different sex and ethnicities. The multidisciplinary research team of this study included both male and female researchers from medical specialities (orthopaedics sports medicine), engineering as well different age categories (junior researchers and professors).

### Statistical analysis

The average score for each of the three criteria was calculated. Additionally, the responses were divided into five different groups based on the level and degree of alignment of the individual grades of the assessors: “fully correct”, “majority correct”, “correct/partial”, “correct/diverging”, and “partially correct/diverging” (Tables 3 and 4). Analysis was conducted using statistical scripts written for the mathematical programming Julia, version 1.8.5.

**Table 4:** Detailed information on responses to questions for target group “non-orthopaedic medical doctor”

Label	Num questions	% questions	Mean correctness	Correctness grading	Example question	ChatGPT response
Fully correct	2	10.0	2.0	2,2,2,2	What strategies should be used to counteract kinesophobia?	To counteract kinesophobia (fear of movement) in patients recovering from knee injuries, a multidisciplinary approach involving physical therapy, psychological support, and patient education is essential. Gradual exposure to activity, progressive strengthening, and proprioceptive training can help improve confidence and reduce fear. Cognitive–behavioural strategies, such as setting achievable goals, relaxation techniques, and addressing catastrophic thinking, can be employed to address the psychological aspects of kinesophobia. Patient education on injury mechanisms, the recovery process, and realistic expectations can also facilitate a more confident return to activities.
Majority correct	11	55.0	1.75	2,2,2,1	What movement patterns and what muscle groups should be trained to avoid anterior cruciate ligament injury and/or anterior cruciate ligament graft rupture?	To prevent ACL injury or graft rupture, training should focus on neuromuscular control, muscle strengthening, and proprioceptive exercises targeting the quadriceps, hamstrings, hip abductors, and gluteal muscles. Emphasis should be placed on functional movements, such as landing and cutting mechanics, to encourage proper knee alignment, joint stability, and load distribution Additionally, plyometric exercises and balance training can help improve dynamic joint stability and reduce injury risk.
Correct/partial	5	25.0	1.5	2,2,1,1	When should osteotomy be performed in the setting of revision anterior cruciate ligament reconstruction?	Osteotomy should be considered in the setting of revision ACL reconstruction when there is significant malalignment, such as varus or valgus deformity, contributing to increased stress on the reconstructed ligament and predisposing the graft to failure. Additionally, osteotomy may be indicated to address bone deficiencies or tunnel malposition from previous surgery. The goal of the osteotomy is to restore normal joint biomechanics, optimize graft function, and improve the likelihood of a successful revision outcome.
Correct/diverging	1	5.0	1.5	2,2,2,0	What are the indications for anterior cruciate ligament repair and when should it be performed and for which patient category?	Indications for ACL repair, as opposed to reconstruction, include proximal ACL avulsion injuries with preserved ligament integrity and good tissue quality, occurring within a short time frame from injury (typically within 2–3 weeks). ACL repair may be suitable for skeletally immature patients, as it preserves the native ligament and avoids potential growth disturbances. However, this procedure is less commonly performed due to the higher risk of failure compared to ACL reconstruction, and its indications are limited to specific patient and injury characteristics.
Partially correct/diverging	1	5.0	1.0	1,1,1,1	What is the most ideal timing for anterior cruciate ligament reconstruction in the setting of concomitant meniscus bucket handle tear and locked knee?	In the setting of an ACL injury with a concomitant bucket handle meniscus tear and locked knee, early surgical intervention is recommended, typically within 2–4 weeks post-injury. Prompt surgery allows for reduction and repair of the displaced meniscus, preserving meniscal tissue, restoring knee function, and preventing further joint damage. Prior to surgery, it is crucial to reduce inflammation and achieve near-normal knee range of motion through appropriate conservative management.

## Results

### High-yield topics within ACL surgery

The average correctness for the responses provided by ChatGPT was calculated to be 1.69 and 1.66 for patients and doctors, respectively (Table 2). Only for 3 out of 20 (15.0%) questions did any of the four orthopaedic sports medicine surgeons judge that the answer was incorrect; however, even for these questions the average correctness score was calculated to be either 1.25 or 1.5. Furthermore, completeness was found to be 1.51 and 1.64 for patients and doctors, respectively, while adaptiveness was calculated to be 1.75 and 1.73 for patients and doctors, respectively. However, the mean completeness was found to be slightly higher when only including responses with a mean correctness score ≥ 1.5 without receiving any score of “0” (Table 2).

#### Patient as target group

A total of 13 (65.0%) of all questions were assessed to be fully correct or majority correct, while only 2 (10.0%) of the questions were assessed to be partially correct or partially correct/diverging (Table 3).

#### Medical non-orthopaedic surgeon as the target group

Among all questions posed to ChatGPT, a total of 13 (65.0%) were deemed fully correct or majority correct, whereas only 1 (5.0%) was considered partially correct or diverging (Table 4).

## Discussion

The main findings of this study indicate that ChatGPT demonstrated the ability to provide overall correct and well-adapted responses in slightly less than two-thirds of the provided prompts, which aligns partially with our hypothesis. However, it is important to note that only 15.0% of the questions were determined to be completely incorrect, emphasizing the importance of good judgement by the user.

ChatGPT’s responses to questions posed by a patient were found to be accurate (fully or majority correct) in 65.0% of the cases. For example, the response to the question “What strategies should be used to counteract kinesophobia?” was graded as “correct” by all reviewers, while the response to the question “What are the most important risk factors for postoperative knee stiffness following ACL reconstruction?” was assessed as correct by a majority of the reviewers. Hence, this suggests that LLMs like ChatGPT may be useful aids for patients preparing for medical consultations, offering an accurate and concise overview of a specific orthopaedic topic, and eliminating the need to conduct a time-consuming literature review.

Most of the partially correct or partially correct/diverging responses were associated with areas that have limited high-quality evidence and where current literature is conflicting. As a result, the risk of misinformation provided by ChatGPT may be higher for topics that lack robust evidence, such as ACL repair. Thus, it is possible that part of the responses provided by ChatGPT may be based on quantity instead of quality of evidence during the pre-training phase and, therefore, it may not be able to differentiate between low- and high-quality data. Nevertheless, these findings are not unexpected, since the LLMs have not been specifically developed to provide expert-level knowledge [12] and have not been fine-tuned into orthopaedic medicine. Given this, the performance of the model may be limited when attempting to acquire expert-level knowledge, indicating a potential for further improvement [19].

The findings of this study also suggest that prompting may have an impact on ChatGPT’s responses. Without a specific prompt, responses were observed longer (1993 words), compared to those generated with prompt 1 (patient; 329 words) or prompt 2 (medical doctor; 552 words), as determined by an average over the first ten responses in our randomized sequence. The absence of a specific prompt might have additionally resulted in a reduced ability to adapt to the target group (patient, non-orthopaedic medical doctor) and subsequently increased the chance of hallucinating. Prompting is therefore essential in decreasing the risk of misinformation when using these models. There is thus a risk that patients will use general models, like ChatGPT, that have not been fine-tuned to the specific domain of orthopaedics and be misinformed prior to meeting an orthopaedic surgeon, since they simply pose their questions and do not know how to prompt the model. The practising clinician should be aware that in addition to patients increasingly making searches on the Internet, they can now likely access more apparently plausible yet misguided arguments from models like ChatGPT.

This study has several limitations. The reliability of the responses generated by ChatGPT was not evaluated, inviting the possibility that responses may have differed if the same question had been asked repeatedly, or if the responses had been ordered differently. Furthermore, ChatGPT-4 as of March 14th, 2023, was used, which is only one type of LLM. Future studies should consider evaluating multiple LLMs to prove a more comprehensive assessment. The three-point response scale used to evaluate responses was not standardized and, therefore, may have limited the objective measurement of correctness, completeness, and adaptability. Thus, the different assessors may have interpreted the scale differently, leading to inconsistencies in the assessment process. To try to mitigate this threat, the same instructions were provided to all assessors and included examples of how to use the scales. Moreover, the four orthopaedic sports medicine surgeons who assessed the responses were not blinded to the fact that the responses were generated by ChatGPT. Therefore, the assessment of the reviewers may have been influenced both by individual bias and their preconceptions about the correctness of LLMs.

While it is important to note that ChatGPT is not a substitute for the expertise of orthopaedic sports medicine surgeons and may struggle to appraise the level of evidence and propagate its responses by struggling with conveying nuances of the English language (distinguishing between “might” and “should”), these models also offer potential as supplementary aids. These models could, for instance, assist in orthopaedic research by analysing text, support clinical practice by summarizing the latest papers for staying up to date, and aid in education by guiding patients through foundational literature prior to their consultations with the orthopaedic surgeon.

## Conclusion

Overall, ChatGPT was successful in generating correct responses in approximately 65% of the cases related to ACL surgery. The findings of this study imply that LLMs offer potential as a supplementary tool for acquiring orthopaedic knowledge. However, although ChatGPT can provide guidance and effectively adapt to diverse target audiences, it cannot supplant the expertise of orthopaedic sports medicine surgeons in diagnostic and treatment planning endeavours, due to its limited understanding of orthopaedic domains and its potential for erroneous responses.

### Supplementary Information

Below is the link to the electronic supplementary material.

Supplementary file1 (DOCX 37 KB)
