# Supplementary material for: Exploring the potential of ChatGPT as a supplementary tool for providing orthopaedic information
Source: Knee Surg Sports Traumatol Arthrosc. 2023 Aug 8;31(11):5190–8. doi: 10.1007/s00167-023-07529-2 (PMC10598178; doi:10.1007/s00167-023-07529-2)
Supplement: Supplementary file 1 — Supplementary file1 (DOCX 37 KB) [file 167_2023_7529_MOESM1_ESM.docx]

**Supplemental Material**

**Table 5. Information on responses to questions for target group “patient, young athlete”.**

| Questions | ChatGPT Response | Mean correctness | Mean Completeness | Mean adaptiveness | Correctness grading | Completeness grading | Adaptiveness grading |
| --- | --- | --- | --- | --- | --- | --- | --- |
| What movement patterns and what muscle groups should be trained to avoid anterior cruciate ligament injury and/or anterior cruciate ligament graft rupture? | To prevent ACL injuries or graft ruptures, focus on training movement patterns like landing, cutting, and pivoting with proper technique. Strengthening key muscle groups such as the hamstrings, quadriceps, glutes, and hip muscles, along with improving core stability and balance, can also help reduce the risk of injury. | 1.75 | 1.5 | 2.0 | 2, 2, 2, 1 | 2, 2, 1, 1 | 2, 2, 2, 2 |
| From a risk factor standpoint what would be the worst  outcome case scenario leading to anterior cruciate  ligament injury? | The worst outcome scenario from a risk factor standpoint would involve a young, highly active athlete with poor neuromuscular control, muscle imbalances, and a history of previous knee injuries. Additionally, the athlete might engage in high-risk movements like pivoting, cutting, or jumping without proper technique, significantly increasing the likelihood of an ACL injury. | 1.75 | 1.5 | 2.0 | 2, 2, 2, 1 | 2, 2, 1, 1 | 2, 2, 2, 2 |
| What is the most ideal timing for anterior cruciate ligament reconstruction in the setting of concomitant meniscus bucket handle tear and locked knee? | In the case of an ACL injury with a concomitant bucket handle meniscus tear and locked knee, surgery should ideally be performed sooner rather than later. Early intervention, within a few weeks of the injury, can help restore knee function, prevent further cartilage damage, and increase the chances of successfully repairing the meniscus. | 2.0 | 1.75 | 2.0 | 2, 2, 2, 2 | 1, 2, 2, 2 | 2, 2, 2, 2 |
| What is the most cost effective treatment for a patient with anterior cruciate ligament tear? If surgery is chosen is there a difference in cost between treatments and what is it? | The most cost-effective treatment for an ACL tear depends on the patient's activity level and lifestyle. For less active individuals, nonoperative treatment with physical therapy and bracing may be more cost-effective. If surgery is required, costs can vary based on graft choice and surgical techniques. Autografts (using patient's own tissue) are usually less expensive than allografts (donor tissue), but the best option should be determined by patient needs and the surgeon's recommendations. | 1.5 | 1.5 | 1.75 | 1, 1, 2, 2 | 1, 1, 2, 2 | 1, 2, 2, 2 |
| When can anterior cruciate ligament injury be treated nonoperatively? | Anterior cruciate ligament (ACL) injuries can sometimes be treated nonoperatively when the injury is minor, the patient has low physical activity demands, and their knee remains stable during daily activities. A combination of physical therapy and a knee brace can help manage these cases. | 1.75 | 1.0 | 1.75 | 1, 2, 2, 2 | 1, 1, 1, 1 | 1,2, 2, 2 |
| What are the indications for anterior cruciate ligament  injuries in children and when should they be treated? | Indications for treating ACL injuries in children include significant knee instability, a desire to return to high-level sports, or associated injuries like meniscus or other ligament tears. Treatment options range from conservative  management with bracing and physical therapy to surgical intervention, such as ACL reconstruction. The timing of treatment depends on the severity of the injury, the child's skeletal maturity, and individual factors discussed with the surgeon. | 1.25 | 1.5 | 1.5 | 0, 1, 2, 2 | 1, 1, 2, 2 | 1, 1, 2, 2 |
| Based on the current knowledge is it worthwhile to biologically enhance anterior cruciate ligament surgery (using a platelet rich plasma, stem cells, microfragmented adipose tissue, plasma clot matrix, fibrin clot, hyaluronic acid)? | Current research shows mixed results regarding the benefits of biologically enhancing ACL surgery with techniques like platelet-rich plasma, stem cells, or other substances. While some studies suggest potential benefits, more research is needed to establish their effectiveness and safety conclusively. | 1.75 | 1.75 | 1.75 | 2, 1, 2, 2 | 2, 2, 2, 1 | 1, 2, 2, 2 |
| What are the indications for use of different anterior cruciate ligament graft types? | The choice of ACL graft type depends on factors like patient age, activity level, personal preference, and surgeon's experience. Common graft options include hamstring autografts (from the patient), bone-patellar tendon-bone autografts, and allografts (from a donor). Young, active patients often receive autografts,  while older, less active patients or those with multiple ligament injuries may receive allografts. | 1.5 | 1.0 | 1.75 | 1, 1, 2, 2 | 1, 1, 1, 1 | 1, 2, 2, 2 |
| When should a lateral augmentation procedure be added to an anterior cruciate ligament reconstruction? | A lateral augmentation procedure, like a lateral extra-articular tenodesis, should be considered in ACL reconstruction when there is significant rotational knee instability, a high risk of graft failure (e.g., young athletes in pivoting sports), or a history of failed primary ACL reconstruction. It can help improve overall knee  stability and reduce the risk of re-injury. | 1.5 | 1.75 | 1.25 | 0, 2, 2, 2 | 1, 2, 2, 2 | 0, 1, 2, 2 |
| When should a medial collateral ligament reconstruction be performed in the setting of anterior cruciate ligament injury? | A medial collateral ligament (MCL) reconstruction should be considered in the  setting of an ACL injury when the MCL tear is severe (grade III) or when the MCL does not heal properly after non-surgical treatment, causing ongoing knee instability or pain despite adequate conservative management. | 1.75 | 1.75 | 2.0 | 2, 2, 2, 1 | 2, 2, 2, 1 | 2, 2, 2, 2 |
| What are the indications for anterior cruciate ligament  repair and when should it be performed and for which  patient category? | ACL repair may be considered for patients with a specific type of injury called a proximal avulsion, where the ligament is torn near its femoral attachment but remains largely intact. This procedure is more suitable for young, active patients who have sustained the injury recently, typically within a few weeks. Early intervention increases the chances of successful healing and avoids further damage to the knee. | 1.25 | 1.0 | 1.5 | 0,1, 2, 2 | 0, 1, 1, 2 | 0, 2, 2, 2 |
| What are the risks and benefits of stump preservation in anterior cruciate ligament reconstruction? | Stump preservation in ACL reconstruction aims to maintain the remaining ACL tissue to potentially improve stability and healing. Benefits include better knee proprioception and faster recovery. However, risks involve possible graft  impingement, inadequate tunnel placement, or complications in revision surgeries. A surgeon will weigh these factors when deciding on the best approach. | 1.5 | 1.75 | 1.75 | 1, 1, 2, 2 | 1, 2, 2, 2 | 1, 2, 2, 2 |
| Where should the femoral tunnel be placed in the setting of anterior cruciate ligament reconstruction? | In ACL reconstruction, the femoral tunnel should be placed at the anatomical footprint of the native ACL on the femur. This is typically located in the posterolateral corner of the femoral intercondylar notch, ensuring proper graft positioning and restoring the knee's normal function and stability. | 2.0 | 1.75 | 1.5 | 2, 2, 2, 2 | 1, 2, 2, 2 | 0, 2, 2, 2 |
| What are the most important risk factors for postoperative  knee stiffness following anterior cruciate ligament  reconstruction? | Key risk factors for postoperative knee stiffness after ACL reconstruction include preoperative knee stiffness, prolonged immobilization, incorrect graft placement, and inadequate rehabilitation. Additionally, factors like infection,  bleeding, or an individual's natural healing response can contribute to stiffness. Proper surgical technique and a well-designed rehabilitation plan can help minimize these risks. | 1.75 | 1.75 | 1.5 | 2, 2, 2, 1 | 2, 2, 2, 1 | 1, 2, 2, 1 |
| What does accelerated rehabilitation mean? | Accelerated rehabilitation refers to a post-surgical recovery program designed to help patients regain strength, mobility, and function more quickly than traditional approaches. It involves earlier initiation of weight-bearing, range of motion exercises, and strength training while closely monitoring the patient's  progress and adjusting the program as needed. | 1.75 | 1.5 | 2.0 | 2, 2, 2, 1 | 1, 2, 2, 1 | 2, 2, 2, 2 |
| What strategies should be used to counteract kinesophobia? | To counteract kinesophobia (fear of movement or re-injury), patients should gradually expose themselves to activities under professional guidance, such as physical therapy. Setting realistic goals, receiving education about the injury and recovery process, and using psychological techniques like relaxation or  visualization can also help manage fear and build confidence. | 2.0 | 1.75 | 2.0 | 2, 2, 2, 2 | 1, 2, 2, 2 | 2, 2, 2, 2 |
| How can patellofemoral problems following anterior  cruciate ligament reconstruction be avoided? | To avoid patellofemoral problems after ACL reconstruction, surgeons should ensure proper graft placement, tensioning, and fixation to avoid altering the knee's biomechanics. Additionally, patients should follow a comprehensive  rehabilitation program that includes quadriceps strengthening, flexibility exercises, and gradual progression of activities to regain proper knee function and minimize patellofemoral issues. | 1.75 | 1.5 | 2.0 | 2, 2, 2, 1 | 1, 2, 1, 2 | 2, 2, 2, 2 |
| What are the most important factors predicting return to  sport following anterior cruciate ligament reconstruction? | Important factors predicting return to sport after ACL reconstruction include completing a comprehensive rehabilitation program, regaining full range of motion, achieving adequate muscle strength and balance, passing functional tests, and having confidence in the knee's stability. Psychological factors, such  as motivation and fear of re-injury, also play a role in successful return to sport. | 1.75 | 1.75 | 2.0 | 1, 2, 2, 2 | 1, 2, 2, 2 | 2, 2, 2, 2 |
| Should the same surgical technique and graft type be  used in revision anterior cruciate ligament reconstruction as in primary anterior cruciate ligament reconstruction? | In revision ACL reconstruction, the choice of surgical technique and graft type may differ from primary reconstruction due to factors like previous graft failure, tunnel placement, or bone loss. Surgeons may use alternative graft sources or techniques to address these challenges and optimize outcomes in revision  cases. | 2.0 | 1.5 | 1.5 | 2, 2, 2, 2 | 1, 1, 2, 2 | 0, 2, 2, 2 |
| When should osteotomy be performed in the setting of  revision anterior cruciate ligament reconstruction? | Osteotomy should be considered in revision ACL reconstruction when there is significant malalignment of the knee joint, such as varus or valgus deformities, which contributes to abnormal loading and instability. Correcting the alignment can help reduce stress on the reconstructed ACL and improve the chances of a successful outcome. | 1.5 | 1.0 | 1.5 | 1, 1, 2, 2 | 1, 1, 1, 1 | 1, 1, 2, 2 |

**Table 6. Information on responses to questions for target group “non-orthopaedic medical doctor”.**

| Questions | ChatGPT Response | Mean correctness | Mean Completeness | Mean adaptiveness | Correctness grading | Completeness grading | Adaptiveness grading |
| --- | --- | --- | --- | --- | --- | --- | --- |
| What movement patterns and what muscle groups  should be trained to avoid anterior cruciate ligament  injury and/or anterior cruciate ligament graft rupture? | To prevent ACL injury or graft rupture, training should focus on neuromuscular control, muscle strengthening, and proprioceptive exercises targeting the quadriceps, hamstrings, hip abductors, and gluteal muscles. Emphasis should be placed on functional movements, such as landing and cutting mechanics, to encourage proper knee alignment, joint stability, and load distribution. Additionally, plyometric exercises and balance training can help improve dynamic joint stability and reduce injury risk. | 1.75 | 1.75 | 1.75 | 2, 2, 2, 1 | 2, 2, 2, 1 | 2, 2, 2, 1 |
| From a risk factor standpoint what would be the worst  outcome case scenario leading to anterior cruciate  ligament injury? | The worst outcome case scenario leading to an ACL injury would involve a combination of intrinsic and extrinsic risk factors. Intrinsic factors may include female sex, generalized ligamentous laxity, increased quadriceps-to-hamstrings strength ratio, and abnormal lower extremity alignment. Extrinsic factors could involve participation in high-risk sports with frequent pivoting, cutting, and jumping, inadequate neuromuscular control, and poor playing surface conditions. An individual with multiple risk factors may be at a significantly higher risk of sustaining an ACL injury. | 1.5 | 1.5 | 1.5 | 1, 1, 2, 2 | 1, 1, 2, 2 | 1, 1, 2, 2 |
| What is the most ideal timing for anterior cruciate ligament  reconstruction in the setting of concomitant meniscus  bucket handle tear and locked knee? | In the setting of an ACL injury with a concomitant bucket handle meniscus tear and locked knee, early surgical intervention is recommended, typically within 2-4 weeks post-injury. Prompt surgery allows for reduction and repair of the displaced meniscus, preserving meniscal tissue, restoring knee function, and preventing further joint damage. Prior to surgery, it is crucial to reduce inflammation and achieve near-normal knee range of motion through appropriate conservative management. | 1.0 | 1.5 | 1.75 | 1, 1, 1, 1 | 1, 1, 2, 2 | 1, 2, 2, 2 |
| What is the most cost effective treatment for a patient with  anterior cruciate ligament tear? If surgery is chosen is  there a difference in cost between treatments and what  is it? | The most cost-effective treatment for ACL tears depends on the patient's individual factors, such as activity level and associated injuries. For some, nonoperative management with rehabilitation may suffice. In surgical cases,  autografts (hamstring, patellar tendon) are generally more cost-effective than allografts due to the latter's higher cost and risk of graft failure. However, cost-effectiveness analyses should consider factors like graft availability, donor  site morbidity, and patient preferences. | 1.5 | 1.5 | 1.5 | 1, 2, 2, 1 | 1, 2, 2, 1 | 1, 2, 2, 1 |
| When can anterior cruciate ligament injury be treated nonoperatively? | Nonoperative treatment of anterior cruciate ligament (ACL) injuries can be considered in patients with partial ACL tears exhibiting minimal instability,  those with low-demand lifestyles or willing to modify their activities, and in  patients with contraindications for surgery. A comprehensive rehabilitation  program focusing on muscle strengthening, proprioceptive training, and  neuromuscular control is essential for successful conservative management. | 1.75 | 2.0 | 2.0 | 1, 2, 2, 2 | 2, 2, 2, 2 | 2, 2, 2, 2 |
| What are the indications for anterior cruciate ligament  injuries in children and when should they be treated? | Indications for ACL reconstruction in children include skeletally mature adolescents with a complete ACL tear and significant functional instability, as well as skeletally immature patients with an ACL tear who have failed nonoperative  management or have concomitant meniscal or chondral injuries. Early surgical intervention may be considered to minimize the risk of secondary joint damage and facilitate return to activity. In skeletally immature patients, modified surgical  techniques, such as physeal-sparing or physeal-respecting procedures, should be utilized to minimize the risk of growth disturbances. | 1.5 | 1.5 | 1.75 | 1, 2, 2, 1 | 1, 2, 2, 1 | 2, 2, 2, 1 |
| Based on the current knowledge is it worthwhile  to biologically enhance anterior cruciate ligament  surgery (using a platelet rich plasma, stem cells,  microfragmented adipose tissue, plasma clot  matrix, fibrin clot, hyaluronic acid)? | Based on current knowledge, the use of biological enhancements in ACL  surgery, such as platelet-rich plasma (PRP), stem cells, microfragmented  adipose tissue, plasma clot matrix, fibrin clot, and hyaluronic acid, remains  controversial. While some studies report positive outcomes regarding graft  healing, reduced inflammation, and improved functional recovery, others  show no significant benefits. More high-quality, randomized controlled trials  are needed to establish the efficacy and optimal application of these biological  adjuncts in ACL surgery. | 2.0 | 2.0 | 2.0 | 2, 2, 2, 2 | 2, 2, 2, 2 | 2, 2, 2, 2 |
| What are the indications for use of different anterior  cruciate ligament graft types? | Indications for different ACL graft types depend on patient factors, surgeon  preference, and availability. Autografts, including bone-patellar tendon-bone  (BPTB) and hamstring tendon grafts, are generally preferred in younger, more  active patients due to lower failure rates and favorable outcomes. BPTB grafts  may be favored in high-demand athletes due to faster incorporation and stronger initial fixation, while hamstring grafts could be preferable for patients concerned about anterior knee pain or donor site morbidity. Allografts are often  considered for older, less active patients or in revision cases with limited autograft options. Synthetic grafts are rarely used due to higher failure rates and are typically reserved for specific cases or as a last resort. | 1.75 | 1.0 | 1.75 | 2, 2, 2, 1 | 1, 1, 1, 1 | 2, 2, 2, 1 |
| When should a lateral augmentation procedure be added  to an anterior cruciate ligament reconstruction? | A lateral augmentation procedure, such as lateral extra-articular tenodesis (LET) or anterolateral ligament (ALL) reconstruction, should be considered in addition to ACL reconstruction in cases with high-grade pivot-shift, ACL revision surgery with persistent rotational instability, hyperlaxity or generalized ligamentous laxity,  and patients involved in high-risk activities predisposing them to rotational injuries. These procedures aim to address anterolateral rotational instability and potentially reduce the risk of graft failure. | 1.75 | 1.75 | 1.75 | 2, 2, 2, 1 | 2, 2, 2, 1 | 2, 2, 2, 1 |
| When should a medial collateral ligament reconstruction  be performed in the setting of anterior cruciate ligament  injury? | Medial collateral ligament (MCL) reconstruction should be considered in the  setting of ACL injury when there is persistent medial knee instability after  nonoperative management of the MCL, typically 6-8 weeks, or in cases of  multiligamentous knee injuries. Concurrent MCL and ACL reconstruction may  be indicated if the MCL injury is a high-grade tear (grade III) or if the MCL has  failed to heal with conservative treatment, resulting in significant valgus instability. | 1.5 | 1.75 | 1.75 | 1, 2, 2, 1 | 2, 2, 2, 1 | 2, 2, 2, 1 |
| What are the indications for anterior cruciate ligament  repair and when should it be performed and for which  patient category? | Indications for ACL repair, as opposed to reconstruction, include proximal ACL avulsion injuries with preserved ligament integrity and good tissue quality, occurring within a short time frame from injury (typically within 2-3 weeks). ACL repair may be suitable for skeletally immature patients, as it preserves the native  ligament and avoids potential growth disturbances. However, this procedure is  less commonly performed due to the higher risk of failure compared to ACL reconstruction, and its indications are limited to specific patient and injury characteristics. | 1.5 | 1.5 | 1.5 | 0, 2, 2, 2 | 0, 2, 2, 2 | 0, 2, 2, 2 |
| What are the risks and benefits of stump preservation in  anterior cruciate ligament reconstruction? | Stump preservation in ACL reconstruction offers potential benefits, including enhanced revascularization, proprioception, and biological integration of the graft. However, risks involve increased technical complexity, potential graft  impingement, and incomplete or insufficient debridement of the injured ACL tissue, which may affect healing and graft incorporation. | 1.75 | 1.75 | 1.75 | 1, 2, 2, 2 | 1, 2, 2, 2 | 1, 2, 2, 2 |
| Where should the femoral tunnel be placed in the setting  of anterior cruciate ligament reconstruction? | In ACL reconstruction, the femoral tunnel should be placed anatomically to  mimic the native ACL footprint and optimize knee biomechanics. This typically corresponds to the posterolateral aspect of the lateral femoral condyle within the "anatomic ACL insertion site" or the "anteromedial bundle insertion site."  The tunnel position can be further refined using surgical landmarks, such as the bifurcate ridge and the lateral intercondylar ridge, or intraoperative imaging to ensure proper tunnel placement and restore native ACL function. | 1.75 | 1.75 | 1.5 | 2, 2, 2, 1 | 2, 2, 2, 1 | 1, 2, 2, 1 |
| What are the most important risk factors for postoperative  knee stiffness following anterior cruciate ligament  reconstruction? | Important risk factors for postoperative knee stiffness following ACL reconstruction include preoperative stiffness, prolonged immobilization,  concomitant meniscal or chondral procedures, and inadequate rehabilitation. Additionally, factors such as infection, hematoma, arthrofibrosis, and surgical complications, like improper graft tensioning or tunnel placement, can contribute to postoperative stiffness. | 1.75 | 1.75 | 1.75 | 1, 2, 2, 2 | 1, 2, 2, 2 | 1, 2, 2, 2 |
| What does accelerated rehabilitation mean? | Accelerated rehabilitation refers to a postoperative recovery protocol that emphasizes early mobilization, weight-bearing, and range of motion exercises to expedite functional recovery following surgery, such as ACL reconstruction. This approach typically involves a progressive, phased program that incorporates neuromuscular control, muscle strengthening, proprioceptive training, and sport-specific exercises. The goal of accelerated rehabilitation is to minimize  joint stiffness, muscle atrophy, and functional limitations while ensuring adequate  graft healing and maintaining patient safety. | 1.75 | 1.75 | 1.75 | 2, 2, 2, 1 | 2, 2, 2, 1 | 2, 2, 2, 1 |
| What strategies should be used to counteract  kinesophobia? | To counteract kinesophobia (fear of movement) in patients recovering from knee injuries, a multidisciplinary approach involving physical therapy, psychological support, and patient education is essential. Gradual exposure to activity, progressive strengthening, and proprioceptive training can help improve  confidence and reduce fear. Cognitive-behavioral strategies, such as setting achievable goals, relaxation techniques, and addressing catastrophic thinking, can be employed to address psychological aspects of kinesophobia. Patient  education on injury mechanisms, the recovery process, and realistic expectations can also facilitate a more confident return to activities. | 2.0 | 2.0 | 2.0 | 2, 2, 2, 2 | 2, 2, 2, 2 | 2, 2, 2, 2 |
| How can patellofemoral problems following anterior  cruciate ligament reconstruction be avoided? | To avoid patellofemoral problems following ACL reconstruction, several strategies can be employed: ensure proper surgical technique, particularly with bone-patellar tendon-bone graft harvesting and tunnel placement; prioritize  early, controlled rehabilitation focusing on quadriceps and hamstring muscle strengthening to maintain balanced muscle forces; address pre-existing patellofemoral issues, such as maltracking or instability, during the surgical  planning; and educate patients on activity modification and appropriate progression of exercises to minimize excessive patellofemoral stress during the postoperative period. | 1.75 | 1.5 | 1.75 | 2, 2, 2, 1 | 2, 2, 1, 1 | 2, 2, 2, 1 |
| What are the most important factors predicting return to  sport following anterior cruciate ligament reconstruction? | The most important factors predicting return to sport following ACL reconstruction include the patient's psychological readiness, functional  performance, graft healing, and adherence to a comprehensive rehabilitation program. Psychological factors, such as self-efficacy, motivation, and fear of reinjury, play a significant  role in successful return to sport. Additionally, achieving adequate muscle strength, neuromuscular control, and sport-specific functional performance is crucial. Ensuring appropriate graft healing and following a structured, progressive rehabilitation protocol also contribute to a successful return to sport. | 1.75 | 1.75 | 1.75 | 2, 2, 2, 1 | 2, 2, 2, 1 | 2, 2, 2, 1 |
| Should the same surgical technique and graft type be  used in revision anterior cruciate ligament reconstruction  as in primary anterior cruciate ligament reconstruction? | In revision ACL reconstruction, surgical technique and graft choice may differ  from primary ACL reconstruction due to factors such as tunnel widening, bone loss, previous graft type, and the cause of primary graft failure. Alternative graft options or graft augmentation may be considered, and the surgical approach may involve addressing bone deficiencies, tunnel malposition, or concomitant  ligamentous injuries. Optimal revision strategies should be determined based on individual patient characteristics, previous surgical history, and specific reasons for graft failure. | 1.75 | 1.75 | 1.5 | 2, 2, 2, 1 | 2, 2, 2, 1 | 1, 2, 2, 1 |
| When should osteotomy be performed in the setting of  revision anterior cruciate ligament reconstruction? | Osteotomy should be considered in the setting of revision ACL reconstruction when there is significant malalignment, such as varus or valgus deformity, contributing to increased stress on the reconstructed ligament and predisposing  the graft to failure. Additionally, osteotomy may be indicated to address bone deficiencies or tunnel malposition from previous surgery. The goal of the osteotomy is to restore normal joint biomechanics, optimize graft function, and  improve the likelihood of a successful revision outcome. | 1.5 | 1.0 | 1.75 | 1, 1, 2, 2 | 1, 1, 1, 1 | 1, 2, 2, 2 |
